# Preface

**Published:** 1820-09-01

**Authors:** 


### Preface

### To No. 2, for SEPTEMBER, 1820.

In this Number of the Journal, the important subject of *Abdominal Inflammation*, particularly as it affects the serous tissues of that division of the body, forms the leading article, in continuation of the *series of illustrations* which it is designed to publish through this medium.

If, in this article, we have ventured to differ from our able countryman, Dr. Abercrombie, on some points of pathology and practice, it is with the profoundest respect for a gentleman whose talents do honour to the most intellectual city, and whose writings irradiate the most instructive Journal in Europe.

*Insanity* is copiously treated of in the Reviews of Drs. Esquirol, Haslam, and Burrows. Dr. Esquirol's Essay on this important disease is little known in this country, though it contains a great mass of valuable matter.

The attention of the faculty is here solicited to a dangerous *puerperal affection*, minutely delineated by that accurate observer, Dr. Marshall Hall,, of Nottingham; while difficult parturition itself, a state often involving the safety both of mother and child, is ably treated of by Dr. Dewees, of America, whose work is, of course, but very little known to the British profession.

Two of the most important subjects which now agitate the medical and surgical world are varioloid diseases and syphilis. These topics are very fully treated of in the present Number of the Journal; and from the excellent works of Mr. Cross and Dr. Hennen, we have collected into a focus, all the prominent features of the questions at issue, and all the practical deductions which can, as yet, be safely drawn from the facts hitherto ascertained.

From the writings of Messrs. Bell, White, Howship, and Shaw, we have selected a mass of the most important facts and highly interesting observations on diseases of the intestines, pelvic viscera, and their outlets, which cannot fail to prove a rich treat to our surgical readers, and a valuable reference in the hurry and anxiety of actual practice.

Such are the leading features of the present number, which we submit, with deference, to that profession whose approbation we are anxious to obtain, but whose indulgence we greatly require, in the execution of a task not unattended with difficulty, as well as labour. It would, at the same time, be doing an injustice to the public, and to our own feelings, did we not here express our grateful sense of the distinguished patronage with which this Journal has been honoured; and which, we are conscious, has been far beyond the deserts of the work. We hope, however, to prove that, in this instance at least, success will have no influence in paralysing those energies, or quenching that zeal which led to its attainment.

